# Supplementary material for: Development of an elderly lifestyle profile: A Delphi survey of multidisciplinary health-care experts
Source: PLoS One. 2020 Jun 2;15(6):e0233565. doi: 10.1371/journal.pone.0233565 (PMC7266339; doi:10.1371/journal.pone.0233565)
Supplement: S1 Data — (DOCX) [file pone.0233565.s001.docx]

Appendix 1. Round questionnaires (English version)

| Lifestyle is an important factor which can affect old adults’ health and quality of life. Therefore, a lot of countries tend to focus healthy lifestyle of older adults as a national health agenda. However, it is evident that lifestyle is very complex concept which is built by multi-domains.  According to the literature, lifestyle is examined by exercise, nutrition, and smoking/alcohol habit. However, this lifestyle concept has some limitations to provide accurate lifestyle profiling for improving health and quality of life of old adults. Hence, our research team developed three lifestyle domains including physical activity, activity participation and nutrition from previous research  According to the three lifestyle domains, we want to develop specific sub-items for elderly’ lifestyle profile. Thus, we would like to hear your professional opinion through this survey.  If you need further information, please don't hesitate to contact us. |
| --- |

| The following is open and closed questions for measuring old adult’s lifestyle based on the previous literature. The following items will be used as evaluation items for physical activity, activity participation, and nutrition among elderly lifestyle. Please answer the each questions or rate the level of suitability for the following evaluation items. |
| --- |

| **NO** | **Closed- ended questions(59 questions)** | **Relevance**  **Strongly irrelevant 1 2 3 4 Strongly relevant** | | | |
| --- | --- | --- | --- | --- | --- |
|  |  | **1** | **2** | **3** | **4** |
| **Ⅰ. Physical activity** | | | | | |
| **1** | How many days did you do aerobic exercise in the last week? |  |  |  |  |
| **2** | On average, how many times did you do aerobic exercise during the day? |  |  |  |  |
| **3** | Do you do as much as you want? |  |  |  |  |
| **4** | How many days did you do anoxic physical exercise in the last week? |  |  |  |  |
| **5** | On average, how many times did you do anoxic physical exercise during the day? |  |  |  |  |
| **6** | Do you do as much as you want? |  |  |  |  |
| **7** | How many days did you do high-intensity physical exercise in the last week? |  |  |  |  |
| **8** | On average, how many times did you do high-intensity physical exercise during the day? |  |  |  |  |
| **9** | Do you do as much as you want? |  |  |  |  |
| **10** | How many days did you do moderate-intensity physical exercise in the last week? |  |  |  |  |
| **11** | On average, how many times did you do moderate-intensity physical exercise during the day? |  |  |  |  |
| **12** | Do you do as much as you want? |  |  |  |  |
| **13** | How many days did you do low-intensity physical activity in the last week? |  |  |  |  |
| **14** | On average, how many times did you do low-intensity physical activity during the day? |  |  |  |  |
| **15** | Do you do as much as you want? |  |  |  |  |
| **16** | How many days did you go walking in the last week? |  |  |  |  |
| **17** | On average, how many times did you walk during the day? |  |  |  |  |
| **18** | Do you do as much as you want? |  |  |  |  |
| **Ⅱ. Activity Participation** | | | | | |
| **19** | Please write out your daily/Saturday/Sunday routine |  |  |  |  |
| **20** | Please write out your Saturday routine |  |  |  |  |
| **21** | Please write out your Sunday routine |  |  |  |  |
| **22** | How many days did you participate in ADL in the last week? |  |  |  |  |
| **23** | On average, how many times did you do ADL per day? |  |  |  |  |
| **24** | Do you participate in ADL as much as you want? |  |  |  |  |
| **25** | How many days did you participate in leisure activities in the last week? |  |  |  |  |
| **26** | On average, how many times did you do leisure activities per day? |  |  |  |  |
| **27** | Do you participate in leisure activities as much as you want? |  |  |  |  |
| **28** | How many days did you participate in social activities in the last week? |  |  |  |  |
| **29** | On average, how much time did you spend on social activities per day? |  |  |  |  |
| **30** | Do you participate in social activities as much as you want? |  |  |  |  |
| **31** | How many days did you participate in productive activities in the last week? |  |  |  |  |
| **32** | On average, how much time did you spend on productive activities per day? |  |  |  |  |
| **33** | Do you participate in productive activities as much as you want? |  |  |  |  |
| **34** | How many days did you participate in education in the last week? |  |  |  |  |
| **35** | On average, how much time did you spend on education per day? |  |  |  |  |
| **36** | Do you participate in education as much as you want? |  |  |  |  |
| **37** | On average, how much time do you spend asleep per day? |  |  |  |  |
| **38** | Do you sleep as much as you want? |  |  |  |  |
| **Ⅲ. Nutrition** | | | | | |
| **39** | Have you eaten rice or grain each day? |  |  |  |  |
| **40** | Have you eaten bread or flour each day? |  |  |  |  |
| **41** | Have you eaten potato or corn each day? |  |  |  |  |
| **42** | Have you eaten meat or chicken breast each day? |  |  |  |  |
| **43** | Have you eaten fish or tofu each day? |  |  |  |  |
| **44** | Have you eaten beans or egg each day? |  |  |  |  |
| **45** | Have you eaten sesame oil each day? |  |  |  |  |
| **46** | Have you eaten butter or margarine each day? |  |  |  |  |
| **47** | Have you eaten cheese each day? |  |  |  |  |
| **48** | Have you eaten seaweed each day? |  |  |  |  |
| **49** | Have you eaten anchovies each day? |  |  |  |  |
| **50** | How much water do you drink per day? |  |  |  |  |
| **51** | How much do you smoke per week? |  |  |  |  |
| **52** | How often do you drink alcohol on average per week? |  |  |  |  |
| **53** | How much do you consume when you drink alcohol each time? |  |  |  |  |
| **54** | How much protein do you think you consumed in the last week? |  |  |  |  |
| **55** | How much carbohydrate do you think you consumed in the last week? |  |  |  |  |
| **56** | How much fat do you think you consumed in the last week? |  |  |  |  |
| **57** | How many vitamins do you think you consumed in the last week? |  |  |  |  |
| **58** | How many minerals do you think you consumed in the last week? |  |  |  |  |
| **59** | How much water do you think you consumed in the last week? |  |  |  |  |

| **3 open-ended questions** | | |
| --- | --- | --- |
| **60** | Are there any items that need to be added to measure the physical activity of the old adults? If Yes, please write on this paper. |  |
| **61** | Are there any items that need to be added to measure the activity participation of the old adults? If Yes, please write on this paper. |  |
| **62** | Are there any items that need to be added to measure the nutrition of the old adults? If Yes, please write on this paper. |  |

Appendix 2. Round questionnaires (Korean version)

**라이프스타일은 고령자의 건강과 삶의 질에 영향을 미치는 중요한 요인입니다. 따라서, 많은 국가들에서는 그들의 국가적 보건정책 아젠다로써, 고령자의 건강한 라이프스타일에 집중하는 경향을 보입니다. 그러나, 기존의 문헌에 따르면, 라이프스타일은 다면적인 요인들로써 성립된 매우 복잡한 개념으로 여겨지고 있습니다.**

**기존의 문헌 고찰에 따르면, 라이프스타일은 주로 운동, 식이, 흡연 및 음주 습관을 통해서만 연구되어 왔습니다. 그러나, 이러한 라이프스타일 개념으로는 고령자의 건강과 삶의 질 증진을 위한 라이프스타일을 프로파일링 하기에 매우 제한적입니다. 따라서, 저희 연구팀은 이전에 선행에서 신체적 활동, 활동 참여와 식이를 포함한 3가지 라이프스타일 구성 영역을 개발 하였습니다.**

**이러한 라이프스타일 영역들에 따라서, 현재 저희 연구팀에서는 각 영역별 하위 평가항목을 개발하고자 본 설문조사를 통해서 현 영역에 전문가이신 귀하의 고견을 듣고자 합니다.**

**만약 본 연구와 관련하여 더 많은 정보가 필요 하시다면, 언제든 연락 주십시오.**

| 다음은 고령자의 건강과 삶의 질을 증진하기 위해 기존의 문헌고찰을 기반으로 고안된 다면적 라이프스타일 측정을 위한 예비 문항 입니다. 다음의 항목들은 고령자의 라이프스타일 중 신체적 활동, 활동 참여와 식이의 평가 문항으로 사용될 예정 입니다. 다음의 평가 문항에 대한 적합도를 평가해 주십시오. |
| --- |

| **NO** | **59개의 폐쇄형 문항** | **적합도**  **매우 부적합 1 2 3 4 매우 적합** | | | |
| --- | --- | --- | --- | --- | --- |
|  |  | **1** | **2** | **3** | **4** |
| **Ⅰ. 신체적 활동** | | | | | |
| **1** | 최근 일주일 동안 당신이 유산소 운동을 한 날은 며칠 입니까? |  |  |  |  |
| **2** | 평균적으로 유산소 운동을 한 시간을 몇 분입니까? |  |  |  |  |
| **3** | 유산소 운동을 원하는 만큼 충분히 하고 있습니까? |  |  |  |  |
| **4** | 최근 일주일 동안 당신이 무산소 운동을 한 날은 며칠 입니까? |  |  |  |  |
| **5** | 평균적으로 유산소 운동을 한 시간을 몇 분입니까? |  |  |  |  |
| **6** | 유산소 운동을 원하는 만큼 충분히 하고 있습니까? |  |  |  |  |
| **7** | 최근 일주일 동안 당신이 고강도 운동을 한 날은 며칠 입니까? |  |  |  |  |
| **8** | 평균적으로 고강도 운동을 한 시간을 몇 분입니까? |  |  |  |  |
| **9** | 고강도 운동을 원하는 만큼 충분히 하고 있습니까? |  |  |  |  |
| **10** | 최근 일주일 동안 당신이 중등도 운동을 한 날은 며칠 입니까? |  |  |  |  |
| **11** | 평균적으로 중등도 운동을 한 시간을 몇 분입니까? |  |  |  |  |
| **12** | 중등도 운동을 원하는 만큼 충분히 하고 있습니까? |  |  |  |  |
| **13** | 최근 일주일 동안 당신이 저강도 운동을 한 날은 며칠 입니까? |  |  |  |  |
| **14** | 평균적으로 저강도 운동을 한 시간을 몇 분입니까? |  |  |  |  |
| **15** | 저강도 운동을 원하는 만큼 충분히 하고 있습니까? |  |  |  |  |
| **16** | 최근 일주일 동안 당신이 걷기 운동을 한 날은 며칠 입니까? |  |  |  |  |
| **17** | 평균적으로 걷기 운동을 한 시간을 몇 분입니까? |  |  |  |  |
| **18** | 걷기 운동을 원하는 만큼 충분히 하고 있습니까? |  |  |  |  |
| **Ⅱ. 활동 참여** | | | | | |
| **19** | 당신의 평일 일과를 써주세요 |  |  |  |  |
| **20** | 당신의 토요일 일과를 써주세요 |  |  |  |  |
| **21** | 당신의 일요일의 일과를 써주세요 |  |  |  |  |
| **22** | 일상생활활동을 한 날은 며칠입니까? |  |  |  |  |
| **23** | 평균적으로 하루에 일상생활활동을 한 시간을 몇 분입니까? |  |  |  |  |
| **24** | 일상생활활동을 원하는 만큼 충분히 하고 있습니까? |  |  |  |  |
| **25** | 여가활동을 한 날은 며칠입니까? |  |  |  |  |
| **26** | 평균적으로 하루에 여가활동을 한 시간을 몇 분입니까? |  |  |  |  |
| **27** | 여가활동을 원하는 만큼 충분히 하고 있습니까? |  |  |  |  |
| **28** | 사회활동을 한 날은 며칠입니까? |  |  |  |  |
| **29** | 평균적으로 하루에 사회활동을 한 시간을 몇 분입니까? |  |  |  |  |
| **30** | 사회활동을 원하는 만큼 충분히 하고 있습니까? |  |  |  |  |
| **31** | 일(노동)을 한 날은 며칠입니까? |  |  |  |  |
| **32** | 평균적으로 하루에 일(노동)을 한 시간을 몇 분입니까? |  |  |  |  |
| **33** | 일(노동)을 원하는 만큼 충분히 하고 있습니까? |  |  |  |  |
| **34** | 교육을 받거나 무언가를 배운 시간은 며칠입니까? |  |  |  |  |
| **35** | 평균적으로 하루에 교육을 받은 시간(혹은 무언가를 배운 시간)을 몇 분입니까? |  |  |  |  |
| **36** | 교육활동을 원하는 만큼 충분히 하고 있습니까? |  |  |  |  |
| **37** | 평균적으로 하루에 수면 시간은 몇 분입니까? |  |  |  |  |
| **38** | 수면을 원하는 만큼 충분히 하고 있습니까? |  |  |  |  |
| **Ⅲ. 식이** | | | | | |
| **39** | 하루에 **쌀 혹은 곡류**를 먹습니까? |  |  |  |  |
| **40** | 하루에 **빵 혹은 밀가루 음식**을 먹습니까? |  |  |  |  |
| **41** | 하루에 **감자 혹은 옥수수**를 먹습니까? |  |  |  |  |
| **42** | 하루에 **살코기 혹은 닭 가슴살을** 먹습니까? |  |  |  |  |
| **43** | 하루에 **생선 혹은 두부를** 먹습니까? |  |  |  |  |
| **44** | 하루에 **콩 혹은 달걀을** 먹습니까? |  |  |  |  |
| **45** | 하루에 **참기름 혹은 들기름**을 먹습니까? |  |  |  |  |
| **46** | 하루에 **버터 혹은 마가린을** 먹습니까? |  |  |  |  |
| **47** | 하루에 **치즈를** 먹습니까? |  |  |  |  |
| **48** | 하루에 **해조류를** 먹습니까? |  |  |  |  |
| **49** | 하루에 **멸치 혹은 잔새우를** 먹습니까? |  |  |  |  |
| **50** | 하룻동안 물은 얼만큼 마십니까? |  |  |  |  |
| **51** | 일주일에 흡연 횟수는? |  |  |  |  |
| **52** | 귀하께서는 일주일에 얼마나 술을 드십니까? |  |  |  |  |
| **53** | 귀하께서 한번에 술을 마시는 양은? |  |  |  |  |
| **54** | 최근 일주일 동안 섭취한 **단백질의 양**은 본인이 생각하기에 어느 정도라고 생각하십니까? |  |  |  |  |
| **55** | 최근 일주일 동안 섭취한 **탄수화물의 양**은 본인이 생각하기에 어느 정도라고 생각하십니까? |  |  |  |  |
| **56** | 최근 일주일 동안 섭취한 **지방의 양**은 본인이 생각하기에 어느 정도라고 생각하십니까? |  |  |  |  |
| **57** | 최근 일주일 동안 섭취한 **비타민 및 무기질의 양**은 본인이 생각하기에 어느 정도라고 생각하십니까? |  |  |  |  |
| **58** | 최근 일주일 동안 섭취한 **미네랄의 양**은 본인이 생각하기에 어느 정도라고 생각하십니까? |  |  |  |  |
| **59** | 최근 일주일 동안 섭취한 **수분의 양**은 본인이 생각하기에 어느 정도라고 생각하십니까? |  |  |  |  |

| **3개의 개방형 문항** | | |
| --- | --- | --- |
| **60** | 고령자의 신체적 활동을 측정하기 위해 추가되어야 할 항목이 있습니까? 만약 있다면 여기에 작성해 주십시오. |  |
| **61** | 고령자의 활동 참여를 측정하기 위해 추가되어야 할 항목이 있습니까? 만약 있다면 여기에 작성해 주십시오. |  |
| **62** | 고령자의 식이를 측정하기 위해 추가되어야 할 항목이 있습니까? 만약 있다면 여기에 작성해 주십시오. |  |
