# Supplementary material for: Development of an elderly lifestyle profile: A Delphi survey of multidisciplinary health-care experts
Source: PLoS One. 2020 Jun 2;15(6):e0233565. doi: 10.1371/journal.pone.0233565 (PMC7266339; doi:10.1371/journal.pone.0233565)
Supplement: Attachment — Submitted filename: Response to Reviewer2.pdf [file pone.0233565.s002.pdf]

Rebuttal letter

Reviewer
PLOS ONE
April 27, 2020

Dear Reviewer,

We thank you and the reviewers for a thorough reading and constructive review of our manuscript and for the opportunity to revise and resubmit. We are pleased to submit the improved research article. Please find below our response to each point raised by the reviewer. We hope that we satisfyingly addressed all of them.

On behalf of my co-author, I appreciate you for your consideration of this resubmission. We look forward to your response.

Sincerely,

Park, Ji-Hyuk, Ph.D. (corresponding author)

otscientist@yonsei.ac.ke

Park, Kang-Hyun, M.S. (first author)

alcsro44@gmail.com

- **Reviewer #2:**

Thank you for the opportunity to review this manuscript outlining the development of a lifestyle profile for older adults. This work has the potential to contribute to the development of a measurement tool to identify to older adults in South Korea who would benefit from a lifestyle modification intervention. While the manuscript is mostly well-written and organised, and is based on a sound review of the literature, I have some concerns with the methods and results. There are also minor concerns relating to the clarity of some sentences, and the terminology used for older adults. These are outlined below and if the authors are able to adequately address these issues the quality of the manuscript will be improved.

Major issues

1. METHODS
• P5, line 97: "… asked to read about the concept of multifaceted lifestyle components" – it would be helpful to the reader to have this information included as a supplementary file.

In order to understand the reader, we provided a supplementary file (the file name is supplementary). This information regarding the concept for multifaceted lifestyle component was developed by our previous research (Park K, Han D, Park H, Ha S, Park. Pilot research for development of multi-faceted lifestyle profile components affecting health and quality of life: Delphi survey. Korean Journal of Occupational Therapy. 2019; 27(3): 105–120).

In order to make clear, we modified the sentence in the manuscript like follow (P5. line 101-106)

"Participants were asked to read about the concept of multifaceted lifestyle components that were developed for creating a lifestyle profile of elders through a file attached survey questionnaire attached to an email (S1 Appendix). In the survey questionnaire, physical activity, nutrition and activity participation are described as key domains of lifestyle based on the literature review and previous study."

• P5, line 99: " … an online document consisting of 65 items" – is this different to the information they were provided about the concept of multifaceted lifestyle components?

Yes, we sent a survey questionnaire which contain two part to the participants. First section in the survey questionnaire demonstrated about the concept of multifaceted lifestyle components that were developed for creating a lifestyle profile based on the previous research (Park et al., 2019). The aim of the first section was to help participants to understand lifestyle components. And second part was consisted of

65 items. Thus, there were two kind of different information included in a survey questionnaire. However, we also agreed that readers might be confused like you, thus, we will provide a survey questionnaire as a supplementary file.

• P5, line 101: "A total of 65 items …" – this is repetitive of line 99. I suggest consolidating the information.

In order to make clear the manuscript, we consolidated the line 99 and line 101 like follow:

"Participants were then shown the next page which was consisted of 65 items: 19 items in the "physical activity" domain, 21 in the "activity participation" domain, and 25 in the "nutrition" domain" (P5. Line 107-109).

• P5, line 103: Figure 1 – Figure 1 outlines "Stage 1, 2, 3" and "First, Second and Third Delphi Survey". However, the text in the Methods uses "Round 1, 2, 3" and "first-round questionnaire" (line 104). It would be easier for the reader to follow if the Figure and the text used the same descriptive wording.

We deeply agreed with your opinion, thus we modified the figure 1 outline form "First, Second, and Third Delphi as well as we modified the manuscript (P5. Line 115).

• P5, lines 110-113: "After finishing their part, the panel participants were e-mailed ……. before starting the study" – this information is already mentioned in the previous paragraph. Suggest consolidating it into the first paragraph of Methods as it provides a clear understanding of what participants were required to complete prior to the first survey/round 1.

As your comment, there were some sentences which should be reorganized. So as to make the manuscript clearly, we consolidated the line 110-113 sentences to the "Delphi method procedures" paragraph (P5, line 112-114).

• P6, line 120: Suggest the use of a different word to 'valence' as the meaning of this word in the sentence is ambiguous; maybe a simpler word that clearly describes what participants were forced to choose would be easier to read?

We apologize for confused you. We looked into the sentence and we found that 'valence' was our mistake. Thus, we change the sentence like following:

"A neutral middle point was not included to force participants to provide their obvious opinions." (P6, line 133)

• P6, lines 123-124: This section is headed Round 3 and mentions that four questions were modified with no questions excluded from Round 2. However, the next sentence then goes back to talking about what happened in Round 2 (three items modified and one added) – which is confusing to me and I wonder whether it would be better mentioned in the Round 2 section? I apologise if I am misunderstanding, but I find it difficult to follow.

We agreed that the sentences from line 123 to 124 could make the reader confused. Therefore, we re-writhed the paragraph. We moved the sentence which are taking about round 2 from the 'Round 3' section to the 'Round 2' section Thus, the final version is like follow:

"**Round 2**

The round 2 survey was developed based on the participants' responses in the first round. The round 2 survey consisted of 59 closed-ended questions grouped into 3 categories (physical activities, activity participation, and nutrition). The participants received the survey via e-mail and were required to score the relevance of each proposed element using a four-point Likert-type scale (strongly relevant, relevant, not relevant, or strongly irrelevant). A neutral middle point was not included to force participants to provide their obvious opinions (Table 1). In round 2, three items were modified and one item was added. Finally, 62 items were composed and grouped into 3 categories

Table 1 is here

Round 3

Round 3 modified four questions and no questions from the second round were excluded. For 65 items, 80% agreement was reached. In the third round, we required the participants to rate the relevance of each item using a four-point Likert-type scale ranging from 1 (strongly irrelevant) to 4 (strongly relevant). The level of consensus was set to 80% of respondents indicating agreement [20]. "

• P7, line 133: "21 panels" – I think this should read 21 panel experts as 21 panels were not used in this study.

To further clarify the sentence, line 133 sentence was revised as follow.

"According to the criteria, the CVR values of all items were set to 0.42 for 21 panel experts in rounds 2 and 3." (P.7. line148)

RESULTS

• P8, line 151: consensus was reached for all 18 physical activity domain items, but Table 1 includes only 6 physical activity items? It is not until the I got to Table 4 that it was clear that you were talking about sub-items of the 6 PA items. I think this could be more clearly explained to avoid confusing the reader.

Like your comment, we agreed that the sentence (line 151) could make the reader confused. As a result of discussing with co-author, we decided to the deleted the item numbers and modified the sentence like below.

"For the physical activity and activity participation categories, consensus was reached on all items" (P.8-7. Line 169-171)

• P10, Table 4: This table outlines the contents of the second Delphi survey and lists questions relating to 59 sub-items. However in the Methods (p6, line 117) the authors state that the 2nd survey consisted of 62 questions. This discrepancy needs to be addressed and clearly explained.

As you pointed out, there was some mistake as we described the results of round 1,2, and 3. Like you said, three sub-items were missing from the Table 4. (Please write out your Saturday routine, please write out your Sunday routine, and have you eaten cheese each day). We corrected Table 4 again (P.10). Thank you for your comment and we also sorry to confuse you.

• P13, line 168: "none of the 62 items analysed" – Table 5 includes 60 questions (as one item was added from the second survey). The discrepancy between 62 and 60 items needs to be addressed, or more clearly explained.

There was a similar mistake above, Thus, we looked at the Table 5, and corrected it. In order to prevent more confusing, we checked every number in the paper.

• P16, Table 6: M, SD and CVR need to be spelled out in full within the table or the abbreviations included as a legend to the table.

In order for the reader to understand the information in the table 6, we added the abbreviations of M, SD and CVR in the table 6.

Minor issues:

1. Terminology for 'older adults'. The authors use different terms throughout the manuscript, including 'older persons', 'older adults' 'elders', 'the elderly'. The generally accepted terms exclude 'elders' and include 'older adults or individuals or people'. This may, however be different in different countries, including for South Korea. I suggest using the accepted appropriate terminology and using it consistently throughout the manuscript.

We also agreed with that using different terms for older adults would be able to make confuse the reader. Hence, we selected 'older adults' as an appropriate terminology and we used it consistently throughout the manuscript instead of 'older persons', 'elders', 'the elderly'.

2. INTRODUCTION

The introduction is clearly and logically organised and well set within the current literature. A few minor suggestions may improve the readability:

• P3, lines 55-56: "Only a few measurements evaluate one's health-related lifestyle. However, they tend to only include ..." – the link between these two sentences is not clear and I suggest combining the sentences to improve the flow, e.g. only a few measurements evaluate one's health-related lifestyle; these tend to only include ....:.

We are appreciated for your suggestion. As you suggested, we combined the sentences as follow.

"Only a few measurements evaluate one's health-related lifestyle; these tend to only include dietary factors, physical exercise, smoking, and/or drinking habits." (P3, line 59)

• P3, line 58: I think what the authors are trying to say is that there has been little specific measurement of individuals' multifacted lifestyles? The way this sentence is currently written is confusing to me and relies on the reader knowing what the multifaceted 'areas' are. A description of multifaceted lifestyle is included in the following paragraph, but some clarity earlier in the introduction would be beneficial.

We also agreed that 'areas' could make the readers confuse thus, we changed the sentence as follow in order to be clear.

"While lifestyles are important to improving the health and well-being of older adults, there has been little specific measurement of individuals' multifaceted lifestyles" (P3, line 61)

3. DISCUSSION AND CONCLUSION
The discussion is well organised and easy to follow. Some minor suggestions include:
• P17, line 190: "crucial factors in assessing the lifestyles …" – it should be clarified that this is according to the expert panel.

In order to be clarified the sentence, we modified it as below,

"According to the expert panel, we also found that physical activities such as "aerobic and anoxic exercise" and "high-, moderate-, and low-intensity activity" are crucial factors in assessing the lifestyles of the older adults." (P.18, line 208-209)

• P17, line 196: Suggested deleting the word 'numerous' and simply state that: research has demonstrated …

As your suggestion, we had made the sentence simpler by deleting the word 'numerous'

"Research has demonstrated that active participation in social activities." (P.18, line 217)

• P17, lines 200-201: "few studies have examined these factors in the elderly with other lifestyle factors" – are you trying to say that few studies have examined these factors combined with other lifestyle factors? If possible this needs to be more clearly stated.

Yes, as you said that, we tried to say that few studies have conducted activity participations combined with other lifestyle factors such as physical activity and nutrition. We realized that this sentence is not clear, so we changed it as below.

"Although there is evidence that engagement in a variety of activities affects health outcomes, few studies have examined, including activity participation along with other lifestyle factors such as physical activity and nutrition." (P.18, line 220-222)

• P.17, lines 202-203: "we were able to assess the life-balance" – you did not assess this, you assessed the opinions of experts and it would be clearer for the reader if you stated that clearly here.

As you pointed out, that sentence was not appropriate because it is not based on the results, thus we decided to delete it so as to avoid misunderstanding of the reader.

4. ABSTRACT

• P.2, line 29: "… questions reconstructed regarding the evaluation items …" – this meaning of this sentence is not clear to me. I am not able to suggest changes, but it should be rewritten to convey its meaning more clearly.

We agreed that the sentence make the readers confused. Thus we deleted some unnecessary words and make it more clear sentence as below.

'The first survey gathered opinions using a mix of open- and closed-ended questions regarding items of the older adults' multifaceted lifestyle profile.' (P2, line 32-33)
